# The Sphingolipid Balance and Endothelial Dysfunction in Lysosomal Storage Diseases: Shared Mechanisms in Gaucher, Niemann–Pick and Fabry Disease

**DOI:** 10.3390/ijms27135972

**Published:** 2026-07-03

**Authors:** Anastasiia Nekrasova, Sergey Kutsev, Alexander Shestopalov

**Affiliations:** 1 Research Centre for Medical Genetics, 1 Moskvorechye St, Moscow 115522, Russia; anastasianekrasova77289@gmail.com (A.N.); kutsev@mail.ru (S.K.); 2 Department of Biochemistry and Molecular Biology, Pirogov Russian National Research Medical University, Ostrovityanova Street, 1, Building 6, Moscow 117513, Russia

**Keywords:** sphingolipids, sphingolipidoses, endothelial dysfunction, S1P, ceramide, Gaucher disease, Fabry disease, Niemann–Pick disease

## Abstract

Endothelial dysfunction underlies many cardiovascular and metabolic diseases. Lysosomal storage disorders, particularly sphingolipidoses, cause intracellular accumulation of specific sphingolipids due to inherited enzyme defects. This review focuses on Gaucher, Niemann–Pick (types A, B, A/B) and Fabry diseases, selected because they exhibit clinically significant cardiovascular manifestations and each accumulates a distinct sphingolipid—glucocerebroside, sphingomyelin, or globotriaosylceramide—allowing comparative analysis of how different metabolic defects converge on similar endothelial phenotypes. We summarize current knowledge on how substrate accumulation disrupts the ceramide/sphingosine-1-phosphate (S1P) rheostat, affecting NO synthase, vascular permeability, inflammation, angiogenesis, autophagy and cell death. Common and disease-specific changes in endothelial morphology and barrier function are discussed. Importantly, direct experimental evidence for endothelial involvement in Gaucher and Niemann–Pick diseases remains scarce; most mechanistic insights derive from non-endothelial cell models, highlighting a significant gap that underscores the need for targeted endothelial studies. Deficiencies of *GBA1*, *SMPD1*, and *GLA* each modulate S1P and ceramide production through distinct pathways, yet all three conditions share similar functional endothelial alterations driven by disrupted sphingolipid homeostasis. Understanding these common mechanisms opens new perspectives for diagnostic biomarkers and therapeutic strategies aimed at restoring sphingolipid balance in the endothelium, though further research is required to validate these findings in endothelial-specific contexts.

## 1. Introduction

The endothelium is an actively functioning “endocrine organ” involved in the regulation of vascular tone, hemostasis, immune response, angiogenesis, and other processes [1]. The endothelium is formed by a monolayer of polarized cells attached to a basement membrane rich in collagen types IV and V, fibronectin and laminin. Adhesion of endothelial cells to the basement membrane is ensured by integrin receptors of various types [2]. In turn, a key component of the apical surface of endothelial cells is the glycocalyx—a multilayer structure with a thickness ranging from several hundred nanometers to 8 μm, consisting of proteoglycans and glycosaminoglycans, mainly heparan sulfate. The functions of the glycocalyx are extremely diverse; for example, it serves as a shear stress sensor between blood and the vessel wall, forms a selective barrier that controls the adhesion and transfer of substances, fluids and cells from the blood through the vascular wall [3]. Disruption of the integrity of the glycocalyx, for example, in the Fabry disease model, is accompanied by increased adhesion of monocytes to the endothelial cell monolayer [4].

## 2. Physiological Functions of the Endothelium and Sphingolipid Signaling

### 2.1. Endothelial Barrier and Transport Functions

The most important function of the endothelium is to create a barrier between the blood and tissues. This function is achieved primarily through a complex system of intercellular contacts: adherens junctions, tight junctions, and gap junctions [5,6]. However, the endothelial barrier is selectively permeable to certain substances and other cells. Barrier permeability is achieved through the restructuring of intercellular endothelial contacts. In the context of sphingolipid metabolism disorders, it is interesting to note that the primary regulation of vascular permeability is realized through receptors for sphingosine-1-phosphate [7], the role of which in endothelial function will be discussed below.

### 2.2. Endothelial Secretory Function and Regulation of Vascular Tone

The endothelium synthesizes a large number of bioactive molecules. These include vasoactive factors, the key one being endothelial NO synthase (eNOS). However, the secretory function of the endothelium is not limited to the synthesis of vasoactive molecules. Weibel-Palade bodies have been shown to contain a wide range of cytokines and chemokines (IL-1, IL-5, IL-6, IL-8, IL-11, and IL-15, granulocyte-macrophage colony-stimulating factor GM-CSF, CCL2, and others) [8].

### 2.3. Hemostasis

Under normal physiological conditions, the endothelium performs an anticoagulant function via several mechanisms, including the binding of heparin sulfate to antithrombin circulating in the blood plasma, the secretion of tissue factor pathway inhibitor (TFPI) and the synthesis of thrombomodulin [9]. In addition to maintaining blood flow, the endothelium plays a pivotal role in thrombus formation under appropriate conditions.

### 2.4. Angiogenesis

Angiogenesis is the process of forming new vessels from existing ones, which enables the expansion and remodeling of the vascular network [10]. The following stages can be distinguished during angiogenesis: endothelial activation, subsequent inhibition of endothelial cell migration and proliferation and restoration of intercellular contacts [10].

### 2.5. Sphingolipids in the Endothelium

**Figure 1:**
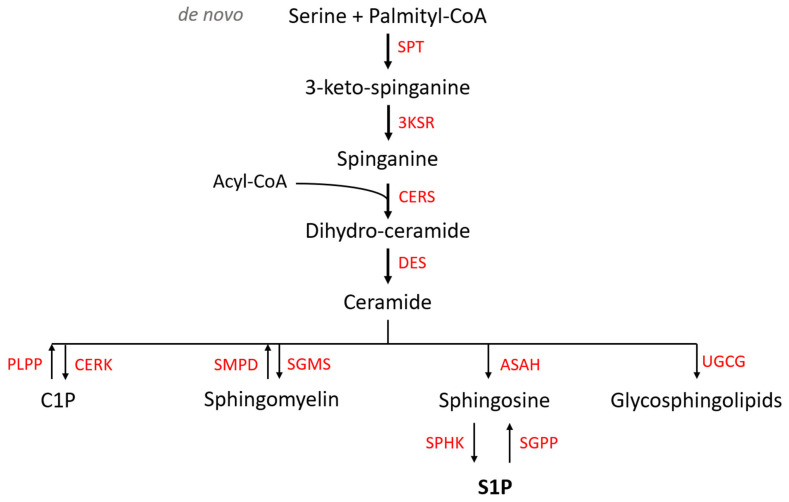
Simplified diagram of ceramide metabolism. SPT—serine palmitoyltransferase, 3KSR—3-ketosphinganine reductase, CERS—ceramide synthase, DES—sphingolipid delta(4)-desaturase, PLPP—phosphate phosphohydrolase, CERK—ceramide kinase, C1P—ceramide-1-phosphate, SMPD—sphingomyelin phosphodiesterase, SGMS—sphingomyelin synthase, ASAH—acid ceramidase, SPHK—sphingosine kinase, SGPP—sphingosine-1-phosphate phosphatase, S1P—sphingosine-1-phosphate, UGCG—ceramide glucosyltransferase.

Sphingolipids (SL) are a class of lipids composed primarily of sphingosine linked by an amide bond to a fatty acid. De novo SL synthesis begins in the endoplasmic reticulum membrane with the condensation of L-serine and a saturated acyl-coenzyme A, typically palmitoyl-CoA, by serine palmitoyltransferase (SPT) (Figure 1). The resulting 3-ketosphinganine is converted to sphinganine by 3-ketosphinganine reductase (3KSR). Sphinganine then undergoes condensation with acyl-CoA by ceramide synthase (CERS) to form dihydroceramide. Dihydroceramide, in turn, is converted to ceramide by dihydroceramide desaturase (DES). The subsequent fate of ceramide involves various pathways. Part of the ceramide is phosphorylated by ceramide kinases to ceramide 1-phosphate. The second pathway of ceramide metabolism is its breakdown into sphingosine, followed by phosphorylation to sphingosine 1-phosphate (S1P). In addition, sphingomyelin and glycosphingolipids can be formed [7].

The diverse functions of sphingolipids in the endothelium have been extensively described in a number of studies [11,12,13].

S1P, the most studied bioactive sphingolipid, plays a crucial role in the regulation of endothelial lining permeability, lymphocyte migration, angiogenesis, and other functions [7]. The main pathway for S1P synthesis in endothelial cells is de novo synthesis: phosphorylation of sphingosine by sphingosine kinases SPHK1 and SPHK2 (Figure 1). Synthesized S1P is transported via SPNS2, entering the bloodstream. Because S1P has hydrophobic properties, it is transported in association with apolipoprotein M in high-density lipoproteins or albumin. It is then able to bind to specific sphingosine 1-phosphate receptors (S1PR) types 1–5 on the surface of endothelial cells and mediate auto- and paracrine effects.

A 2000 study first demonstrated S1P’s role in barrier function, showing that S1PR1-deficient mice died from increased vascular permeability [14]. Additional knockout of S1PR2 and S1PR3 led to an increase in this phenotype. However, there are studies showing that knockout of S1PR1 did not lead to death of experimental mice but caused an increase in vascular permeability [15]. It has also been shown that incubation of endothelial cells with exogenous S1P leads to an increase in endothelial barrier properties [16]. Active signaling through S1PR leads to rapid activation of Rac GTPase, which causes a reorganization of the cortical actin network and an improvement in barrier properties. Another study demonstrated that activation of the small GTPases Rac and Rho is necessary for S1P-induced adherens junction assembly [17]. However, Feng M, et al. [18], who studied the effect of the SPHK1/S1P pathway on the blood-brain barrier, obtained conflicting data. It was shown that cerebral hemorrhages in humans and mice are accompanied by increased SPHK1 expression and S1P plasma levels. Furthermore, SPHK1 inhibition resulted in decreased hematoma and cerebrospinal fluid volume, stabilization of tight junctions between endothelial cells and a decrease in endothelial transcytosis. Thus, the role of S1P in maintaining the endothelial barrier is controversial and requires further investigation.

The involvement of S1P in the regulation of vascular tone has also been described in the literature. S1P is primarily involved in mechanotransduction signaling in response to changes in blood flow. For example, mice with knockout of the sphingosine 1-phosphate transporter gene SPNS2 exhibited reduced levels of blood flow-mediated vasodilation [19]. The role of S1P in regulating vascular tone was also studied by Kerage D, et al. [20], who demonstrated activation of endothelial NO synthase (eNOS) in response to intravascular administration of S1P. eNOS, in turn, stimulates the production of NO, which is a vasodilator. However, some S1P can penetrate the tunica media, where it causes smooth muscle contraction and vasoconstriction. These data indicate a bidirectional effect of S1P on the regulation of vascular lumen.

In addition, S1P is involved in other endothelial functions, such as thrombus formation, migration of other cell types through the endothelial lining and angiogenesis. S1P has been shown to modulate PAR1-AP-mediated platelet aggregation [21]. S1P also plays a role in lymphocyte migration and, consequently, the development of the immune response. The higher S1P concentration in blood compared to lymph promotes S1PR1-dependent lymphocyte migration into the blood or lymph [22]. Sphingosine signaling in the endothelium during angiogenesis is described in detail by Argraves KM, et al. [23].

The significance of glycosphingolipids (GSLs) in the regulation of endothelial functions merits particular consideration. GSLs play a dual and complex role in the endothelium, acting simultaneously as structural components of the cell membrane and as regulatory molecules. As part of the glycocalyx, they participate in cell adhesion and immune responses [24]. Various representatives of GSLs have a specific effects on endothelial function; for example, accumulation of ganglioside GM1 on the surface of senescent endothelial cells promotes the development of insulin resistance [25], and sulfoglucuronosyl paragloboside (SGPG) acts as a critical regulator of cell survival and maintenance of the integrity of the blood-brain barrier [26]. Under inflammatory conditions, cytokine-induced SGPG expression increases, triggering a cascade of signaling pathways that can lead to endothelial cell apoptosis. Impaired general sphingolipid metabolism, in particular excessive accumulation of glycosphingolipids and ceramides, is closely associated with endothelial dysfunction, contributing to the development of atherosclerosis and other cardiovascular diseases [27]. Interestingly, hemodynamic stress can induce a protective rewiring of sphingolipid metabolism in the endothelium, shifting the balance towards S1P and providing an atheroprotective effect [28]. Thus, glycosphingolipids act as the most important mediators in signal transmission, determining the functional state of the endothelium in normal and pathological conditions.

## 3. The Concept of Endothelial Dysfunction (ED) and the Sphingolipids’ Role in ED Development

Endothelial dysfunction (ED) encompasses a variety of pathological processes. ED underlies many cardiovascular [29,30] and metabolic diseases [31]. The causes of dysfunction are extremely diverse and range from hypertension, diabetes and hyperlipidemia to factors such as smoking and obesity. Aging, inflammation, oxidative stress, impaired autophagy and other factors contribute significantly to ED development. Despite the diversity of ED causes, common stages of its development can be identified. Early events typically include endothelial activation, remodeling of intercellular contacts, increased vascular permeability and impaired endothelial secretory activity (e.g., abnormal NO secretion). As the dysfunction progresses, there is an increase in the prothrombotic state, followed by thrombus formation, atherosclerosis, aging and ultimately endothelial cell death [32].

Thus, a number of the most common disturbances characteristic of endothelial dysfunction can be identified: impaired autophagy and state of the lysosomal compartment, oxidative stress, acquisition of a senescent phenotype and—in the context of sphingolipidoses—alterations in the sphingolipid rheostat [27]. In this paper, the term “sphingolipid rheostat” refers to the regulated balance of sphingolipid anabolism and catabolism, primarily involving two interconvertible signaling lipids: ceramide and S1P. Figure 1 provides a comprehensive schematic overview of the broader sphingolipid metabolic network. Within this network, the rheostat axis comprises the central interconversions involving ceramide, sphingosine and S1P (the pathways linked to CERK, ASAH, SPHK, SGPP, etc.), while the upstream de novo branch serves as the metabolic source for ceramide production. Ultimately, these disturbances lead to functional changes, such as increased adhesion or impaired NO synthesis.

Alterations in the sphingolipid rheostat underlie ED in coronary atherosclerosis. Certain lipids, such as ceramide 18:0, ceramide 16:0 and ceramide 24:1, have been shown to participate in inflammatory, thrombotic and low-density lipoprotein (LDL)-mediated atherogenesis pathways [33]. Conversely, S1P levels are reduced in patients with coronary artery disease [28]. Obesity is associated with suppressed de novo synthesis of ceramides and S1P, leading to the activation of proinflammatory genes [11].

Impaired autophagy has been widely described in diseases such as atherosclerosis and diabetes mellitus [34,35]. It is worth noting that patients with diabetes (mainly type 2) exhibit dyslipidemia, characterized by triglyceride accumulation, low HDL levels and high LDL levels due to impaired lipophagy [34]. In lymphatic endothelial cells, impaired lipophagy stimulates lipid droplet accumulation, as well as decreased mitochondrial ATP synthesis and leads to angiogenesis defects [36]. It has also been shown that mechanical stress, such as that caused by portal hypertension, can lead to excessive activation of autophagy, causing ferroptotic cell death [37]. The state of the lysosomal compartment also changes in endothelial dysfunction. It has been shown that in endothelial dysfunction induced by incubation with advanced glycation end products, the first stage is characterized by lysosome permeabilization, resulting in the accumulation of GM3, GD1b and GT1b gangliosides and apoptosis. Cells that survive the initial apoptotic crisis enter a state of stress-induced senescence after 3–5 days. This is manifested by cell enlargement, cell cycle retardation and the appearance of a senescence marker, senescence-associated β-galactosidase [38].

The acquisition of a senescent phenotype by endothelial cells exacerbates some diseases [39]. For example, it has been shown that elderly patients with metabolic dysfunction-associated steatohepatitis (MASLD) have a more severe course of the disease and a higher risk of cirrhosis compared to younger individuals [40,41]. This is due to the fact that sinusoidal liver endothelial cells begin to acquire a senescent phenotype quite early, associated with oxidative stress, triglyceride accumulation, inductive changes, etc. Transplantation of senescent endothelial cells into healthy mice induces bone loss and metabolic dysfunction leading to obesity, while elimination leads to a reversal of the proinflammatory environment and an improvement in the metabolic state [42].

The final stage of endothelial dysfunction is cell death. Various regulated cell death pathways may be involved in the development of endothelial dysfunction, for instance, apoptosis (including autophagy-mediated), necroptosis, pyroptosis, entosis, ferroptosis, ferroautophagy, parthanatosis, netotic cell death, lysosome-dependent cell death, alkaliptosis, oxaptosis, cuproptosis and panoptosis [43].

## 4. Endothelial Dysfunction in Gaucher Disease

**Figure 2:**
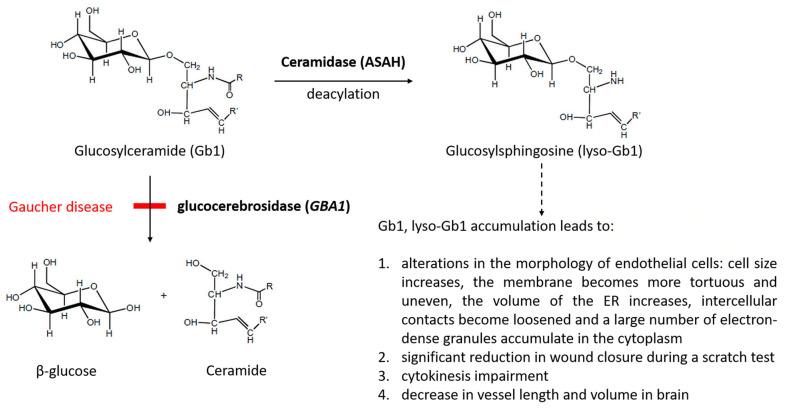
In Gaucher disease, glucocerebrosidase (*GBA1*) deficiency—most frequently resulting from the N370S mutation [46]—leads to the lysosomal accumulation of glucosylceramide (Gb1) and glucosphingosine (lyso-Gb1) within endothelial cells. This pathological buildup subsequently induces morphological alterations [47], impairs cell migration [44], disrupts cytokinesis [44], and reduces both the volume and length of blood vessels in vivo [44].

Gaucher disease (GD) is a rare disorder belonging to a group of lysosomal storage disorders. It is caused by a mutation in the glucocerebrosidase gene *GBA1* (Figure 2). This mutation leads to the accumulation of glucosylceramide (Gb1), its deacetylated form glucosphingosine (lyso-Gb1) and other substrates in lysosomes [44]. The incidence of Gaucher disease is 1 in 50,000–100,000 live births, but in the Ashkenazi Jewish population this rate is significantly higher—approximately 1 in 850 [45].

Traditionally, several subtypes of Gaucher disease are distinguished depending on the time of onset and clinical manifestations. The adult type (GD-1) is characterized by hepatosplenomegaly, bone deformities and cytopenias. The childhood type (GD-2) has the most severe manifestations, including hepatosplenomegaly and significant neurological impairment. Patients with the juvenile type (GD-3) may experience symptoms characteristic of the first two types, albeit less severe. Life expectancy in GD-3 is higher compared to GD-1 and GD-2 [48]. The most characteristic cardiovascular manifestations in Gaucher disease are restrictive cardiomyopathy [49], leading to heart failure, thrombocytopenia and, less commonly, anemia and leukopenia [50]. Patients with Gaucher disease are also noted to have a tendency to subcutaneous hemorrhages, which are usually associated with thrombocytopenia and coagulation disorders [45]. However, this may be directly related to the state of the endothelial barrier, although research on this topic is extremely limited.

### 4.1. Alterations in the Sphingolipid Rheostat in Gaucher Disease

The absence of a functional glucocerebrosidase enzyme leads to changes in the sphingolipid rheostat of cells. Primarily, there is an accumulation of the main substrate of glucocerebrosidase, glucosylceramide (Gb1) [51]. Glucosylceramide can be deacetylated to lyso-Gb1, whose concentration is also elevated in GD [52]. In addition, increased levels of ceramide, trihexosylceramide (THC), GM3, GM2, GM1 and a significant decrease in the level of GT gangliosides in the spleen, liver and brain are noted [44,53]. It is worth noting that increased ceramide levels are not an obvious manifestation of GD. Since ceramide is a product of glucosylceramide catabolism, decreased ceramide levels are expected with GBA1 deficiency. This may suggest de novo activation of the ceramide synthesis pathway or other pathways.

**Figure 3:**
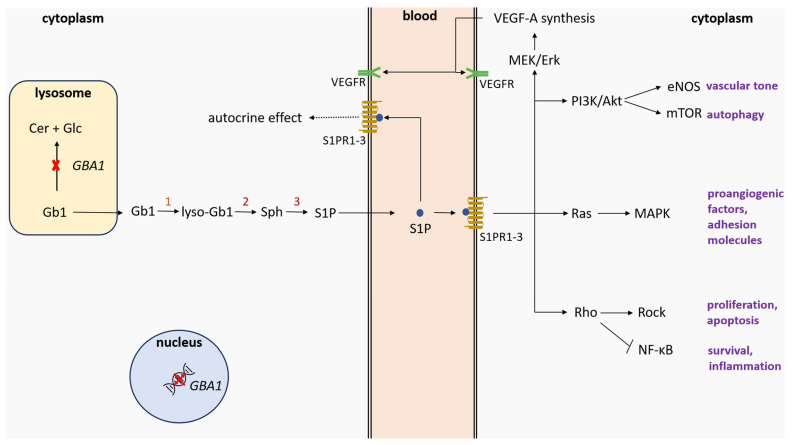
Proposed molecular mechanism of glucosylceramide (Gb1) metabolism and subsequent signaling in Gaucher disease. In the absence of functional glucoceramidase (*GBA1*) [46], Gb1 is cleaved by ceramidase to lyso-Gb1 (glucosphingosine) [52]. Lyso-Gb1, in turn, is presumed to be converted to sphingosine by the non-lysosomal glucoceramidase (*GBA2*). Sphingosine is then phosphorylated by sphingosine kinases to S1P, which is transported into the bloodstream via SPNS2 [19]. S1P regulates cellular functions through auto- and paracrine mechanisms, activating signaling pathways responsible for the reorganization of intercellular junctions and the cytoskeleton, and the inflammatory response in endothelial cells, influencing permeability and immune cell penetration during inflammation [54,55]. 1, ceramidase; 2, non-lysosomal glucosylceramidase (*GBA2*); 3, sphingosine kinase (SPHK1/SPHK2); Sph, sphingosine; S1P, sphingosine-1-phosphate, S1PR 1-3-sphingosine 1-phosphate receptor 1-3.

An increase in S1P concentrations has also been observed in Gaucher disease models [54]. In parallel, a paper by Salah et al. [55] demonstrated a significant increase in VEGF concentrations in the plasma of patients with Gaucher disease compared to controls. Based on these findings, the following molecular mechanism can be proposed (Figure 3). In the absence of functional β-glucocerebrosidase (*GBA1*), Gb1 cannot be broken down into glucose and ceramide in lysosomes. However, it can be deacylated in the cytoplasm to lyso-Gb1 by ceramidase. Lyso-Gb1 can then be converted to sphingosine (Sph) by non-lysosomal glucocerebrosidase (*GBA2*). Sphingosine is then phosphorylated by sphingosine kinases (Sphk1/Sphk2) to S1P. S1P exits the cell and enters the blood, where it can exert auto- and paracrine effects on other cells. Therefore, the elevated S1P levels observed in the study [44] may be associated with activation of the compensatory Gb1 metabolism pathway.

In neighboring cells, S1P binds to its receptors (S1PR1, S1PR2 and S1PR3 are predominantly present on the surface of endothelial cells). This can trigger various signaling pathways. For instance, the PI3K/Akt pathway, on the one hand, leads to increased synthesis of the vasodilator eNOS and, on the other hand, regulates the mTOR complex. In this context, the elevated VEGF levels in patients with Gaucher disease are understandable [55]. Immunoprecipitation and immunocytochemistry have shown that S1PR1 and VEGFR2 form physical and functional complexes on the cell surface [56]. Activated PKC-β1 phosphorylates ERK1/2, which in turn induces the synthesis of VEGF-A. VEGF-A enters the blood plasma and affects cells through auto- and paracrine mechanisms. By binding to its receptors (mainly VEGFR1 and VEGFR2), VEGF-A, for example, triggers the PI3K/Akt pathway, leading to eNOS synthesis. However, convincing data on changes in VEGFR expression on the surface of endothelial cells in Gaucher disease are lacking. Assuming that VEGFR levels were unchanged, this may explain why the mouse model used in [44] showed a reduction in vessel length and volume, as angiogenesis was not induced.

### 4.2. Direct Evidence from Endothelial Cells in Gaucher Disease

Endothelial cell changes in GD are observed at both the morphological and functional levels. It is currently known that Gaucher disease is associated with an increase in the number of capillaries in the dermis and changes in capillary architecture. Ultrastructural analysis of skin biopsies from GD patients revealed morphological changes in endothelial cells: cell size increases, the membrane becomes more tortuous and uneven, the volume of the ER increases, intercellular contacts become loosened and a large number of electron-dense granules accumulate in the cytoplasm [47].

In vitro evidence of altered endothelial function includes studies of endothelial cell motility and angiogenesis. For instance, a scratch test on a monolayer of HUVEC cells incubated with exogenous lyso-Gb1 demonstrated a significant reduction in wound closure and cytokinesis impairment, which may lead to impaired angiogenesis and ED. This is consistent with an in vivo decrease in vessel length and volume in the brain of model mice [44]. The mechanisms by which deacylated monohexosylceramide molecules influence angiogenesis remain unclear. β-glucosylsphingosine and β-galactosylsphingosine are known to stimulate phospholipase A2 (PLA2) activity, which in turn synthesizes lysophosphatidylcholine, an antiangiogenic factor. These molecules can also inhibit the synthesis of the proangiogenic factor thrombin [44]. Endothelial cell death likely occurs through the activation of RIPK3 and MLKL, two major effectors of necroptosis, as in macrophages in GD [57].

### 4.3. Indirect Evidence and Hypotheses from Non-Endothelial Cells in Gaucher Disease

Impaired autophagy is one of the key mechanisms for the development of pathology in GD [58]. In Gaucher disease (GD), the autophagic pathway is disrupted at multiple levels, including early induction, lysosomal integrity, and autophagic flux, although evidence of lysosome-dependent cell death is lacking. Glucocerebrosidase-deficient neuronal cells show impaired TFEB expression—a master regulator of autophagy and lysosomal genes—reducing their autophagic response capacity from the outset. Concomitantly, lysosomal dysfunction is manifested by conflicting changes in LAMP proteins: while some studies report an increase in LAMP2 in neurons and astrocytes from mice [59,60], others describe decreased LAMP1 and LAMP2 levels in cells derived from induced pluripotent stem cells from GD patients, suggesting depletion of the lysosomal compartment and a potential block in the terminal organelle [60]. Moving downstream, autophagic flux is further impaired by decreased levels of the Atg5/12 complex, which impairs the lipidation of LC3-I to LC3-II and thus interferes with normal autophagosome formation [61]. This defect is accompanied by accumulation of p62, a classic marker of impaired degradation, in neurons from mice with Gaucher disease [59,60]. However, data on the LC3-II/LC3-I ratio remain inconsistent, with both increases [60] and decreases [61] reported, possibly reflecting differences in blockade depending on cell type or time course. Collectively, these observations indicate that autophagy is blocked at early and mid-stages (induction and elongation) in conjunction with lysosomal failure, but direct evidence linking these defects to lysosome-dependent cell death is lacking. Moreover, similar disturbances in endothelial cells have not been investigated, and the question of whether the observed autophagic/lysosomal changes ultimately trigger a specific cell death pathway remains open and requires further study.

## 5. Endothelial Dysfunction in Niemann—Pick Disease Types A, B and A/B

**Figure 4:**
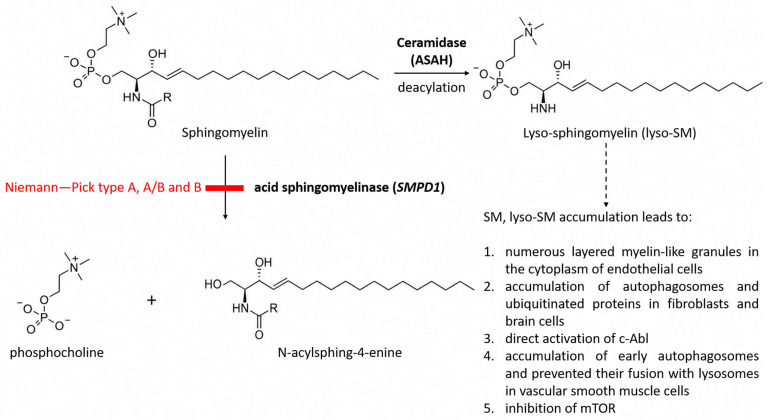
Deficiency of functional acid sphingomyelinase (*SMPD1*) results in the lysosomal accumulation of sphingomyelin and its metabolic derivatives across a range of cell types. This lipid storage burden subsequently triggers a cascade of cellular pathologies, including the deposition of electron-dense granules within the endothelial cytoplasm [63], the progressive build-up of autophagosomes and ubiquitinated protein aggregates in fibroblasts and neural cells [64], direct activation of the c-Abl kinase [65], impaired fusion of early autophagosomes with lysosomes in vascular smooth muscle cells [66], and mTOR suppression [67].

Niemann—Pick disease is a lysosomal storage disorder associated with mutations in the *SMPD1* gene encoding acid sphingomyelinase. Deficiency of this enzyme leads to intracellular accumulation of sphingomyelin and its aberrant metabolites (Figure 4). It is worth noting that Niemann—Pick disease types A, A/B and B are associated with abnormalities in the *SMPD1* gene, while type C is associated with a mutation in the *NPC1* or *NPC2* gene and is not discussed in this review [62].

The main symptoms of Niemann—Pick disease are hepatosplenomegaly, liver cirrhosis, thrombocytopenia, neurological manifestations (dysphagia, ataxia, dystonia), depression, psychosis and many others [62]. Cardiovascular manifestations are somewhat more pronounced in Niemann-Pick disease type B and include coronary artery disease, bleeding and heart failure [68].

### 5.1. Alterations in the Sphingolipid Rheostat in Niemann—Pick Disease

The primary substrate that accumulates in acid sphingomyelinase deficiency (ASMD) is sphingomyelin. Other sphingomyelin-derived lipids, such as ceramide and its derivatives—sphingosine, lyso-SM, lyso-SM-509, glycosphingolipids and bis(monoacylglycerol)phosphate—also accumulate [69]. Concurrently, increased levels of cholesterol, LDL and triglycerides are observed, while HDL level decreases [70]. The mechanism underlying cholesterol accumulation in lysosomes, in addition to that of sphingomyelin, is not fully understood but may be related to sphingomyelin’s high affinity for cholesterol. This leads to impaired cholesterol efflux from lysosomes and subsequent cholesterol accumulation [71].

### 5.2. Direct Evidence from Endothelial Cells in Niemann—Pick Disease

Endothelial dysfunction in NPD has been described very fragmentarily. Morphological changes in NPD type A/B include numerous layered myelin-like granules in the cytoplasm of endothelial cells [63]. A similar picture is observed in NPD type C [72]. However, these data do not provide a comprehensive picture or elucidate the molecular mechanisms of endothelial dysfunction. Systematic studies assessing the functional state of the endothelium, its role in systemic manifestations of the disease (e.g., pulmonary and cardiovascular damage) and its contribution to overall pathogenesis are virtually nonexistent. It remains unclear whether the observed endothelial changes are primary in the pathogenesis or a secondary consequence of metabolic disturbances. Thus, the existing knowledge gap requires comprehensive studies aimed at a comprehensive assessment of the structural and functional state of the endothelium in various forms of ASMD, which could reveal new therapeutic targets and treatment approaches.

### 5.3. Indirect Evidence and Hypotheses from Non-Endothelial Cells in Niemann—Pick Disease

A state of “autophagic stress” is characteristic of various cell types in NPD. In sphingomyelinase deficiency, there is an enhancement of early autophagy induction through inhibition of mTOR, which dephosphorylates TFEB and promotes its nuclear translocation to activate autophagic gene expression [67]. However, the main pathological defect appears to be at later stages, as evidenced by the accumulation of autophagosomes and ubiquitinated proteins in NPD type A fibroblasts and brain cells [64], and in NPD type B lymphocytes, where lipophagy and mitophagy are also impaired [73]. The impairment of this flux is associated with the activation of c-Abl kinase, which regulates TFEB, p73, HDAC2, and APP, and the authors suggest that sphingomyelin and other lipids directly trigger c-Abl [65]. Furthermore, in vascular smooth muscle cells, ASMD induces early accumulation of autophagosomes and prevents their fusion with lysosomes [66]. The late-stage block is further supported by observations in ASMD neurons that exhibit normal levels of early autophagy proteins (Atg5/Atg12 complex) but elevated levels of LC3-II, LAMP1, and LAMP2, along with enlarged autophagosomes and lysosomes [64]. The low substrate degradation capacity may be explained by impaired lysosomal protease activity; indeed, sphingomyelinase deficiency has been shown to cause lysosomal permeabilization and leakage of cathepsin B, a cysteine protease, into the cytosol [64]. However, no articles have addressed autophagy impairment specifically in endothelial cells in NPD types A and B, underscoring the need for further research to clarify which stages of the pathway are primarily affected and whether these defects contribute to cell death.

Despite the above-mentioned changes, sphingomyelinase deficiency has been shown to have an anti-apoptotic effect on endothelial cells [74]. Similar results were obtained in earlier studies [75]. These contradictory results require clarification.

## 6. Endothelial Dysfunction in Fabry Disease

**Figure 5:**
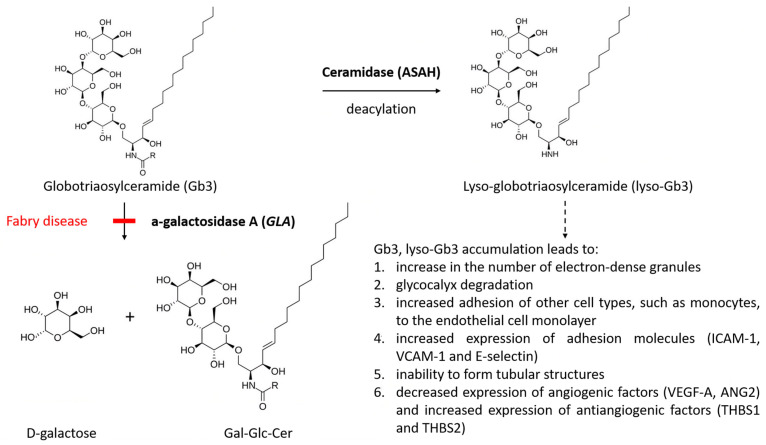
Deficiency of α-galactosidase A (GLA) leads to lysosomal accumulation of globotriaosylceramide (Gb3) and its derivatives in endothelial cells, which in turn elicits a cascade of phenotypic alterations: increased electron-dense granules [77,78], disruption of the glycocalyx [4], enhanced monocyte adhesion [4], upregulated expression of ICAM-1, VCAM-1, and E-selectin [79], impaired tube formation [80], reduced levels of pro-angiogenic factors (VEGF-A, ANG2), and elevated anti-angiogenic mediators (THBS1, THBS2) [80].

Fabry disease is a rare X-linked disorder caused by a mutation in the α-galactosidase A (*GLA*) gene. This enzyme plays a key role in sphingolipid metabolism, hydrolyzing the α(1 → 4) bonds of globotriasylceramide Gb3 (Figure 5). Gb3 is an important component of cell membranes and is involved in intracellular signaling and intercellular communication. Deficiency of functional α-galactosidase A leads to the accumulation of Gb3, as well as its deacetylated form, lyso-Gb3, in various tissues and organs [76].

Clinical manifestations of Fabry disease include neurological pain, acroparesthesia, angiokeratomas, anhidrosis and cornea verticillate [81]. Cardiovascular manifestations of the disease deserve special attention. These include left ventricular hypertrophic cardiomyopathy, less commonly right ventricular hypertrophy, fibrosis, heart failure, arrhythmia and angina pectoris [82,83]. Endothelial dysfunction has been described in more details than in Gaucher disease.

### 6.1. Alterations in the Sphingolipid Rheostat in Fabry Disease

In FD, changes in sphingolipid levels are observed directly in cells, as well as in blood plasma. Primarily, the substrate of α-galactosidase A, Gb3, accumulates [84] and this accumulation occurs at the level of the endoplasmic reticulum membrane [85]. Moreover, the accumulation of Gb3 can induce the NF-κB signaling pathway, its downstream targets [84] and other signaling pathways. Interestingly, the endothelial dysfunction observed in FD is primarily due to Gb3 accumulation and not α-galactosidase A deficiency [86]. However, Gb3 can be metabolized by deacetylation to lyso-Gb3. Consequently, lyso-Gb3 concentrations are also elevated in FD [87]. Additionally, glycosphingolipid levels, such as galabiosylceramide Gb2, may be elevated [88]. Lyso-Gb3 and S1P levels are significantly elevated in the plasma of patients with Fabry disease [87,89].

### 6.2. Direct Evidence from Endothelial Cells in Fabry Disease

Morphological changes in endothelial cells in Fabry disease have long been described. A study by Nepomnyashchikh GI, et al. [77], revealed the accumulation of electron-dense granules, fibrillar structures and specific granules with a regular architecture in the cytoplasm of endothelial cells. A similar phenotypic picture was shown in the work of Najafian B, et al. [78]. Structural changes in cells also affect the glycocalyx of endothelial cells. In cells with the *GLA* gene knockout, a decrease in the content of L-fucose and N-acetylglucosamine—the main components of the glycocalyx—and, consequently, glycocalyx thinning were shown [4]. Moreover, exposure of endothelial cells to exogenous lyso-Gb3 leads to the same result. Glycocalyx degradation causes increased adhesion of other cell types, such as monocytes, to the endothelial cell monolayer. These findings align with previous results showing increased expression of adhesion molecules (such as ICAM-1, VCAM-1 and E-selectin) in *GLA* deficiency [79].

The pathological effect is not only caused by sphingolipids (Gb3, lyso-Gb3), but also by the misfolded enzyme accumulating in the ER lumen. Elevated levels of mutant α-galactosidase A lead to chronic ER stress and a response to misfolded proteins [90].

These phenotypic and molecular changes lead to functional alterations in the endothelium. *GLA*-deficient endothelial cells are unable to form tubular structures in Matrigel, a classic model of angiogenesis [80]. Furthermore, decreased expression of angiogenic factors (VEGF-A, ANG2) and increased expression of antiangiogenic factors (THBS1 and THBS2) have been demonstrated. This indicates active inhibition of angiogenesis in FD. Moreover, the observed decrease in VE-cadherin and eNOS may cause increased vascular permeability and the appearance of multiple angiokeratomas. Aberrant NO synthase synthesis, as well as increased production of reactive oxygen species (ROS), lead to oxidative stress [91]. *GLA* deficiency has also been shown to increase the expression of cyclooxygenase-2 (COX-2), a proinflammatory factor that increases inflammation in the vascular region [91]. These data are consistent with the results of a histological study demonstrating the presence of perivascular inflammation in FD [92].

Taken together, direct data obtained in endothelial cells indicate that FD is characterized by complex endothelial pathology, including structural remodeling, glycocalyx degradation, a transition to a pro-adhesive and pro-inflammatory phenotype and suppression of angiogenesis. However, the precise molecular cascades linking Gb3/lyso-Gb3 accumulation to specific endothelial responses (e.g., impaired autophagy, abnormal cell death pathways) remain poorly understood and most mechanistic insights have been obtained in non-endothelial cell models. Furthermore, the clinical application of potential ED biomarkers, such as VEGFα, is limited by the lack of validated FD-specific marker panels and large prospective studies linking these biomarkers to long-term cardiovascular outcomes.

### 6.3. Indirect Evidence and Hypotheses from Non-Endothelial Cells in Fabry Disease

Impaired autophagy is a key driver of endothelial dysfunction in Fabry disease, with different molecular steps differentially affected. Lyso-Gb3 triggers early autophagy induction in ARPE-19 cells (primarily as ER-selective autophagy) [93], whereas in HEK293T cells expressing mutant GLA, an increased LC3-II/LC3-I ratio and elevated p62 levels support initial pathway activation [94]. However, the fact that autophagosome numbers remain unchanged over time in these mutant cells suggests a subsequent impairment of autophagic flux, specifically pointing to a block in autophagosome—lysosome fusion [94]. This fusion defect is further emphasized by lysosomal dysfunction, as GLA deficiency results in increased LAMP-2 protein and more LysoTracker-positive dots, reflecting lysosomal proliferation and likely impaired acidification [94]. In addition, lyso-Gb3 induces inflammation and necroptosis in an autophagy-dependent manner, and conditioned medium from ARPE-19-treated cells may provoke necroptosis and an inflammatory phenotype in HUVECs, suggesting that prolonged lysosomal stress and autophagy blockade, together with secreted factors from the microenvironment, converge to promote cell death [93].

## 7. Therapeutic Implications and Future Directions

Understanding ED mechanisms and sphingolipid roles may advance diagnostic and therapeutic approaches for sphingolipidoses. Current therapeutic strategies include enzyme replacement therapy (ERT), substrate reduction therapy (SRT) and pharmacological chaperones.

ERT remains the mainstay for Gaucher disease type 1 and Fabry disease and is under evaluation for Niemann-Pick disease [95,96,97]. ERT has been shown to reduce lysosomal substrate load in various cell types and can partially reverse some vascular abnormalities. However, ERT has limited efficacy in reversing established fibrosis and is ineffective at crossing the blood-brain barrier, limiting its use in neuropathic forms.

Substrate reduction therapy (SRT) employs small molecules that inhibit glycosphingolipid synthesis, thereby reducing the production of accumulating substrates. Miglustat and eliglustat are approved for Gaucher disease [98,99], and venglustat is being investigated in Fabry disease [100]. SRT can be used as monotherapy or in combination with ERT, and may offer advantages in terms of oral administration and tissue penetration. However, its efficacy in reversing endothelial dysfunction has not been systematically evaluated.

Pharmacological chaperones (e.g., migalastat for Fabry disease) bind and stabilize mutant forms of α-galactosidase A, enhancing its residual activity [101]. This oral therapy is effective only in patients with amenable GLA mutations and has shown beneficial effects on cardiac and renal parameters, but data on endothelial outcomes remain scarce.

The central role of the ceramide/S1P balance in endothelial homeostasis makes it an attractive therapeutic target. As discussed above, all three diseases exhibit alterations in S1P and ceramide levels, suggesting that pharmacological modulation of this rheostat could restore endothelial function.

Selective SPHK1/SPHK2 inhibitors could reduce excessive S1P signaling, which contributes to vascular permeability, inflammation, and aberrant angiogenesis in some contexts. Given the dual role of sphingosine-1-phosphate in endothelial function, a more precise approach would be to use S1P receptor modulators. Drugs such as fingolimod (FTY720) or more selective S1PR modulators can precisely regulate endothelial barrier function [102]. Given that S1PR1 activation enhances barrier integrity, while S1PR2/3 can promote permeability, agonists or antagonists that selectively target these receptors could be developed to restore normal endothelial function without systemic immunosuppression.

Since ceramide accumulation is observed in Gaucher disease and Niemann-Pick disease, inhibitors of ceramide synthesis, such as myriocin [103], or activators of ceramide degradation (acid ceramidase enhancers) may restore balance. Alternatively, drugs that promote the conversion of ceramide to S1P (via SPHK) or non-toxic metabolites may be useful, but excessive S1P formation should be avoided.

Whereas these diseases are accompanied by impaired autophagy, autophagy stimulators (e.g., rapamycin, trehalose, or TFEB activators) can restore lysosomal clearance and reduce endothelial stress. Similarly, given that necroptosis is involved in the development of Gaucher disease and Fabry disease, RIPK1/RIPK3 inhibitors may prevent endothelial cell death and preserve vascular integrity.

Given the multifactorial nature of ED, combining ERT with sphingolipid rheostat modulation may be more effective than monotherapy.

## 8. Conclusions

Thus, as demonstrated above, lysosomal storage diseases—Gaucher, Fabry and Niemann-Pick diseases—are characterized by ED. This dysfunction manifests itself in both morphological and functional changes in the endothelial lining, which are summarized in Table 1. The morphological changes in these storage diseases are quite similar: an increase in cell size is observed, as well as the formation of numerous granules in the cytoplasm. These changes are explained by the physical accumulation of substrates in lysosomal and other cellular compartments.

However, no direct data exist on autophagy, lysosomal status, or cell death types in endothelial cells for these diseases. In other cell types, blockade of autophagy at early and later stages, accumulation of lysosomes and autophagolysosomes and death by necroptosis may be observed (Table 2). Further studies are essential to bridge the prevailing gaps and establish a fundamental understanding of ED development in these sphingolipidoses.

Future research should prioritize several specific areas. First, the development of endothelial-specific disease models—for instance, endothelial cells derived from iPSC and harboring patient-specific mutations. This will allow direct study of the effects of sphingolipid accumulation and testing of therapeutic agents. Second, because the endothelium of different vessels is highly heterogeneous, single-cell vascular profiling technologies (e.g., single-cell RNA sequencing and spatial transcriptomics) are needed to identify vulnerable subpopulations within different vascular beds that may be preferentially affected in each disease. Third, comprehensive lipidomic analyses of plasma, endothelial cells and tissue biopsies are needed to quantify the full spectrum of sphingolipid species and identify disease-specific lipid signatures that correlate with endothelial function outcomes. Finally, to implement these results into clinical practice, allowing for early diagnosis, monitoring of disease progression and evaluation of the effectiveness of treatment, careful validation of potential biomarkers such as lyso-Gb1, lyso-Gb3, S1P, and VEGF and others is essential.

## 9. Literature Search Strategy

This is a narrative review. A literature search was conducted in PubMed, Scopus and Web of Science databases using combinations of the following keywords: “sphingolipids,” “sphingolipidoses,” “endothelial dysfunction,” “Gaucher disease”, “Fabry disease”, “Niemann-Pick disease”, “sphingosine-1-phosphate”, “ceramide”, “autophagy” and “angiogenesis.” The search period covered publications from 2000 to 2025. Nevertheless, our selection process prioritized the most current scholarship, focusing specifically on articles published within the last five years. Articles were included if they addressed endothelial dysfunction, sphingolipid metabolism or vascular pathology in the context of lysosomal storage diseases. References were also screened for additional relevant studies.

## Figures and Tables

**Table 1:** Summary of morphological and molecular changes in the endothelium in Gaucher disease, Niemann—Pick disease (types A, B, A/B) and Fabry disease.

	Gaucher Disease	Niemann—Pick Disease A, B and A/B	Fabry Disease
Main accumulated substrate	Gb1, lyso-Gb1 [44,52,53,54]	sphingomyelin, lyso-SM, lyso-SM-509, Gb1, GM2, GM3 [69,70,71]	Gb3, lyso-Gb3, Gb2 [84,85,87]
Endothelial cell morphology	cells are enlarged, membrane is folded, apical parts of cells protrude into the vessel lumen, intercellular contacts are partially loosened [47]	numerous membranous cytoplasmic bodies [63]	cells are enlarged, with many electron-dense granules in the cytoplasm; glycocalyx is thinned [4,77,78]
Endothelial barrier permeability	loosening of intercellular contacts [47]	no data found	VE-cadherin decreased [91]
VEGF level	increased [55]	no data found	decreased [80]
S1P level	increased [54]	no data found	increased [89]
Angiogenesis	presumably decreased [44]	no data found	decreased [80]

**Table 2:** Summary of molecular changes in the non-endothelial cells (fibroblasts, lymphocytes, etc.) in Gaucher disease, Niemann—Pick disease (types A, B, A/B) and Fabry disease.

	Gaucher Disease	Niemann—Pick Disease A, B and A/B	Fabry Disease
Autophagy	blocked at early stages (Atg5/Atg12 decreased) [61]	late-stage autophagy blockade: mTOR inhibition; LC3-II increased [64,67]	late-stage blockade: LC3-II/LC3-I and p62 increased; accumulation of autophagosomes [94]
Lysosomes	conflicting data (LAMP1, LAMP2 increased/decreased) [59,60]	LAMP1, LAMP2 increased [64,67]	LAMP2 increased; LysoTracker-positive cells increased [94]
Cell death	necroptosis [57]	antiapoptotic effect of ASMD [74,75]	necroptosis [93]

## Data Availability

No new data were created or analyzed in this study.

## Abbreviations

The following abbreviations are used in this manuscript:

3KSR	3-ketosphinganine reductase
Akt	Protein kinase B
ASAH	Acid ceramidase
ASMD	Acid sphingomyelinase deficiency
C1P	Ceramide-1-phosphate
CCL2	C-C motif chemokine ligand 2
CERK	Ceramide kinase
CERS	Ceramide synthase
COX-2	Cyclooxygenase-2
DES	Sphingolipid delta(4)-desaturase
ED	Endothelial dysfunction
EDHF	Endothelial-derived hyperpolarizing factor
eNOS	Endothelial NO synthase
ERK1/2	Extracellular signal-regulated kinases 1 and 2
FD	Fabry disease
Gb1	Glucosylceramide/Glucocerebroside
Gb2	Galabiosylceramide
Gb3	Globotriaosylceramide
GBA1	Glucocerebrosidase
GBA2	Non-lysosomal glucocerebrosidase
GD	Gaucher disease
GLA	α-galactosidase A
GM-CSF	Granulocyte-macrophage colony-stimulating factor
HDL	High-density lipoproteins
HUVEC	Human umbilical vein endothelial cells
ICAM-1	Intercellular adhesion molecule 1
IL	Interleukin
iPSC	Induced pluripotent stem cells
LAMP1	Lysosome-associated membrane glycoprotein 1
LAMP2	Lysosome-associated membrane glycoprotein 2
LDL	Low-density lipoprotein
lyso-Gb1	Glucosylsphingosine
lyso-Gb3	Globotriaosylsphingosine
MASLD	Metabolic dysfunction-associated steatotic liver disease
MLKL	Mixed lineage kinase domain-like protein
mTOR	Serine/threonine-protein kinase mTOR
NF-κB	Nuclear factor kappa-light-chain-enhancer of activated B cells
NO	Nitric oxide
NPD	Niemann-Pick disease
PI3K	Phosphatidylinositol 3-kinase
PLA2	Phospholipase A2
PLPP	Phosphate phosphohydrolase
Rac	Ras-related C3 botulinum toxin substrate
Rho	Ras homolog family member
RIPK3	Receptor-interacting serine/threonine-protein kinase 3
ROS	Reactive oxygen species
S1P	Sphingosine-1-phosphate
S1PR1	Sphingosine 1-phosphate receptor 1
S1PR2	Sphingosine 1-phosphate receptor 2
S1PR3	Sphingosine 1-phosphate receptor 3
SGMS	Sphingomyelin synthase
SGPP	Sphingosine-1-phosphate phosphatase
SL	Sphingolipids
SMPD	Sphingomyelin phosphodiesterase
SMPD1	Acid sphingomyelinase
Sph	Sphingosine
SPHK	Sphingosine kinase
SPNS2	Sphingosine-1-phosphate transporter SPNS2
SPT	Serine palmitoyltransferase
TFEB	Transcription factor EB
TFPI	Tissue factor pathway inhibitor
THC	Trihexosylceramide
UGCG	Ceramide glucosyltransferase
VCAM-1	Vascular cell adhesion molecule 1
VEGF	Vascular endothelial growth factor
VEGFR1	Vascular endothelial growth factor receptor 1
VEGFR2	Vascular endothelial growth factor receptor 2
